# Dissecting the T Cell Response: Proliferation Assays *vs.* Cytokine Signatures by ELISPOT

**DOI:** 10.3390/cells1020127

**Published:** 2012-05-10

**Authors:** Donald D. Anthony, Kimberly A. Milkovich, Wenji Zhang, Benigno Rodriguez, Nicole L. Yonkers, Magdalena Tary-Lehmann, Paul V. Lehmann

**Affiliations:** 1 Department of Medicine, Case Western Reserve University, Cleveland, OH 44106, USA; E-Mails: kimmilk@wowway.com (K.A.M.); benigno.rodriguez@case.edu (B.R.); nicole.yonkers@novartis.com (N.L.Y.); 2 Department of Pathology, Case Western Reserve University, Cleveland, OH 44106, USA; E-Mails: magda.tary-lehmann@immunospot.com (M.T.-L.); paul.lehmann@immunospot.com (P.V.L.); 3 The Veterans Administration Medical Center, Cleveland, OH 44106, USA; 4 University Hospitals of Cleveland, Cleveland, OH 44106, USA; 5 Divisions of Infectious and Rheumatic Diseases, Cleveland, OH 44106, USA; 6 Case Western Reserve University, Cleveland, OH 44106, USA; 7 Cellular Technology Limited, Cleveland, OH 44122, USA; E-Mail: wenji.zhang@immunospot.com; 8 The Center for AIDS Research, Cleveland, OH 44106, USA

**Keywords:** T cell, proliferation, cytokine, ELISPOT

## Abstract

Chronic allograft rejection is in part mediated by host T cells that recognize allogeneic antigens on transplanted tissue. One factor that determines the outcome of a T cell response is clonal size, while another is the effector quality. Studies of alloimmune predictors of transplant graft survival have most commonly focused on only one measure of the alloimmune response. Because differing qualities and frequencies of the allospecific T cell response may provide distinctly different information we analyzed the relationship between frequency of soluble antigen and allo-antigen specific memory IFN-γ secreting CD4 and CD8 T cells, their ability to secrete IL-2, and their proliferative capacity, while accounting for cognate and bystander proliferation. The results show proliferative responses primarily reflect on IL-2 production by antigen-specific T cells, and that proliferating cells in such assays entail a considerable fraction of bystander cells. On the other hand, proliferation (and IL-2 production) did not reflect on the frequency of IFN-γ producing memory cells, a finding particularly accentuated in the CD8 T cell compartment. These data provide rationale for considering both frequency and effector function of pre-transplant T cell reactivity when analyzing immune predictors of graft rejection.

## 1. Introduction

Host T cells that recognize allogeneic MHC molecules on transplanted tissue are mediators of chronic graft rejection [1,2,3]. In addition to genetically encoded differences in MHC molecules, somatic/environmental events may also shape the magnitude and quality of the graft specific T cell repertoire [2,3]. Efforts to select organs that will be well tolerated by the host need to both consider donor-recipient MHC match, and recipient T cell repertoires prior to transplantation. It is difficult to find organs that are matched for all HLA-alleles and even small variations in MHC subtypes can trigger classic T cell mediated graft rejection. Efforts to predict rejection on the basis of proliferative mixed lymphocyte reactions have been unsuccessful. However, a different method of analysis of recipient pre-transplant donor-specific T cell reactivity has shown promise. In particular, the frequency of graft specific IFN-γ producing memory T cells before transplantation appears to be predictive of graft rejection [1,4,5,6,7]. Such memory T cells are thought to result from priming with cross reactive antigens. If for example, the recipient happens to have recently undergone an infection such as influenza, the frequencies of influenza-specific memory cells will be high, constituting up to 10% of all T cells present in this subject. If these influenza-specific T cells are cross reactive against donor A tissue (but not against donor B tissue), donor A’s (but not donor B’s) organ will be rejected in an accelerated manner. As an example, T cells primed to a specific pathogen influence the outcome of allograft survival [2,8]. Therefore a more refined analysis of pre-transplant alloreactivity may allow for identification of clinically predictive information.

One critical factor in determining the outcome of a T cell response in general is clonal size; that is the frequency of antigen specific T cells within the re-circulating T cell pool [9]. Another critical factor is the primed *vs.* naïve state of antigen-specific T cells. Naïve T cells are readily amenable to pharmacologic immune modulation, such as treatment with cyclosporine and FK506, while memory cells are rather resistant to standard immune suppressive therapy. Therefore, a high number of alloreactive naïve T cells capable of mounting a strong proliferative response may have a fundamentally different implication for transplantation medicine than do a high number of alloreactive memory T cells that may or may not proliferate efficiently.

Cytokine signatures permit a distinction between naïve and memory T cells. Memory cells engage in the production of cytokines such as IFN-γ within 20 h after antigen challenge, while naïve T cells must first undergo proliferation and differentiation before they can express such cytokines [10,11,12]. Also a subset of uncommitted memory cells has been described that produces IL-2 and can differentiate into either IFN-γ or IL-4 producing (Th1 or Th2-like) cells [13]. Both the frequency and the memory state of T cells can be readily measured by *ex vivo* short term ELISPOT assays. Because IL-2 is an autocrine growth factor, the ability of naïve or memory T cells to produce IL-2 is likely related to the proliferative capacity of the T cells. Finally, it has been generally assumed that (allo) antigen-induced proliferation measures the expansion of the antigen-specific T cells, without a major bystander reaction, while indeed the production of cytokines such as IL-2 have the potential to trigger proliferation in bystander cells, blurring identification of clonal size of antigen-specific T cells in some cases, and potentially influencing the function of T cells present in the analysis.

In this study we utilized peripherally derived human lymphocyte populations to analyze the relationship between frequency of antigen and allo-antigen specific, cytokine secreting, memory CD4 or CD8 T cells, and their proliferative capacity. Bystander cell proliferation was also taken into account. The results show that proliferative responses primarily reflect on IL-2 production by antigen-specific T cells. Additionally, proliferating cells in such assays entail a considerable fraction of non-T bystander cells. Proliferation (and IL-2 production) did not reflect on the frequency of IFN-γ producing memory cells. These data support the concept that a more detailed analysis of pre-transplant T cell reactivity using refined approaches that take into account frequency of alloantigen-specific memory cells is appropriate for identifying immunologic predictors of allograft survival.

## 2. Materials and Methods

### 2.1. Cell Isolation

Participants were adult healthy individuals. All study subjects provided written informed consent, and all studies were performed with approval of the institutional review board for human studies at University Hospitals of Cleveland. PBMC, CD3- depleted PBMC (>97% CD3- cells; RosetteSep CD3 depletion reagent; StemCell Technologies, Vancouver BC, Canada), CD3/56 depleted PBMC (>95% CD3/56- cells; RosetteSep reagent), CD4 T cells (negative selection method, RosetteSep reagent), and CD8 T cells (negative selection method using R&D systems, Inc., Minneapolis MN, USA) were freshly prepared from peripheral blood specimens.

### 2.2. Soluble Antigen Specific T Cell IFN-γ and IL-2 ELISPOT Assay

PBMC were plated (3 × 10^5^ cells/well), in the presence (in duplicate) or absence (in triplicate) of protein antigen (Mumps, Biowhittaker, Walkersville, MD, USA; 1:8, Candida, Greer Laboratories, Lenoir NC USA, 10 ug/mL) or CD8 peptide antigen (EBV BMLF-1 GLCTLVAML, EBNA3a RLRAEAQVK, or EBNA3b IVTDFSVIK Panatech, Tubingen, Germany at 2 ug/mL). 96 well ELISPOT cell cultures were incubated for 20 h at 37 °C, developed and analyzed as previously described [14,15,16,17].

### 2.3. Allogeneic T Cell Cytokine Producing Assay

Three hundred thousand CD3 depleted or CD3/CD56 depleted PBMC stimulators, prepared from the blood of healthy controls, and 300,000 PBMC, CD4, or CD8 T cell allogeneic healthy control responder populations were co-cultured in duplicate in 96 well plates at 37 °C in complete RPMI medium (Gibco BRL, Grand Island NY) with 1% penicillin-streptomycin, 1% L‑glutamine, and 5% human AB serum (Gemini Bio-Products, Woodland CA). Cultures were carried out for 20 or 72 h. For 20 h cultures, plates were pre-coated with cytokine capture antibody to perform ELISPOT analysis. For 72 h cultures cells were transferred to pre-coated ELISPOT plates during the final 20 h. The plates were then developed and analyzed as described previously for single color and 2-color spots [16].

Briefly, ELISPOT plates (Whatman Inc., Clifton NJ, USA) were coated overnight with capture cytokine antibodies diluted in sterile PBS: for IFN-γ detection, mouse anti-human IFN-γ (clone 2G1, Endogen, Wolburn MA) at 4 μg/mL; for IL-2 detection, anti-human IL-2 (clone 5334, R&D systems, Inc., Minneapolis MN, USA) at 8μg/mL. For 2-color assays wells were incubated with 50 μL IFN-γ coating antibody 10 minutes prior to addition of 50 μL IL-2 coating antibody. After overnight coating antibody incubation, plates were blocked for 1 h with PBS+1%BSA, then washed three times with PBS. After culture, cells were discarded, and plates were washed three times with PBS followed by four washes with PBS with 0.025% Tween (PBST). Detection antibodies diluted in PBST with 1% BSA were: anti-human IFN-γ FITC (clone 4S.B3, eBiosceince, San Diego CA , USA) at 3 μg/mL for IFN-γ, anti-human IL-2 biotin (clone B33-2, Endogen) at 0.06 μg/mL for IL-2. For 2-color ELISPOT 50 μL IL-2 detection was added first, followed 10 minutes later by 50 μL IFN-γ detection antibody, then incubated overnight at 4 °C. The plates were washed three times with PBST and incubated 2 h at room temperature with 100 μL streptavidin HRP (Dako, Carpenteria CA) at 1:2000 for IL-2 detection, or 100 μL anti-FITC streptavidin APC at 1:500 (Dako). For 2-color experiments both reagents were added at the same time. Plates were washed, then developed with 3-Amino-9-ethyl carbazole reagent (Pierce Chemical Co., Rockford IL) for IL-2 detection, or Vector Blue (Vector laboratories, Burlingam CA, USA) for IFN-γ detection. For 2-color experiments blue reagent was added first until blue spots developed (10–20 min), followed by plate washing and red spot development. The resulting red and blue spots were counted using a computer assisted ELISPOT image analyzer (Cellular Technologies Limited, Cleveland OH), designed to detect 2-color spots using predetermined criteria based on size, shape, and colorimetric density.

### 2.4. ^3^H-Thymidine Incorporation Proliferation Assay

For analysis of PBMC, CD4 or CD8 T cell proliferation by ^3^H-thymidine incorporation, 5 day cultures were performed in duplicate. During the final 16 h of culture ^3^H-thymidine (0.5 μCi/well) was added, and incorporation was measured on day 5.

### 2.5. Proliferation by CFSE Dye Dilution Method

For proliferation analysis by CFSE method, PBMC (5 × 10^6^) were washed once with 2.5 mL PBS with 0.1% BSA, then suspended in 250μL PBS with 0.1% BSA (BSA concentration determined for lot of CFSE). Cells were stained for 10 min at 37 °C, then 5 mL 10% FBS was added and cells were incubated 5 min on ice. Labeled cells were spun, then resuspended in culture media at 0.5 mL/million cells. Stimulator (CD3/56 depleted PBMC, 1 × 10^6^) and allogeneic responder CFSE labeled PBMC (1 × 10^6^) cells were cultured in 24 well plates 37 °C in 5% CO2 5 days.

Cells were stained with anti-CD8 APC and anti-CD4 PE (BD, Mountainview CA) 20 min at 25 °C, washed in PBS with 0.01% BSA, fixed in 1% paraformaldehyde, then stored at 4 °C until analysis. Flow cytometric analysis was performed on a FACScalibur (Becton Dickinson) flow cytometer with Cell Quest Software (Becton Dickenson). Lymphocytes were identified by forward and side light scatter, and the frequencies of CD4 or CD8 T cells with CFSE dye dilution was determined.

### 2.6. Statistical Analysis

Nonparametric Spearman’s test was used to explore correlations. Group comparisons were analyzed by Mann-Whitney’s U test. All analyses were done using SPSS version 11.0 (Chicago, IL).

## 3. Results

### 3.1. Antigen-Specific CD4 and CD8 Memory Cells Display Dissociated Production of IL-2 and IFN-γ

**Figure 1:**
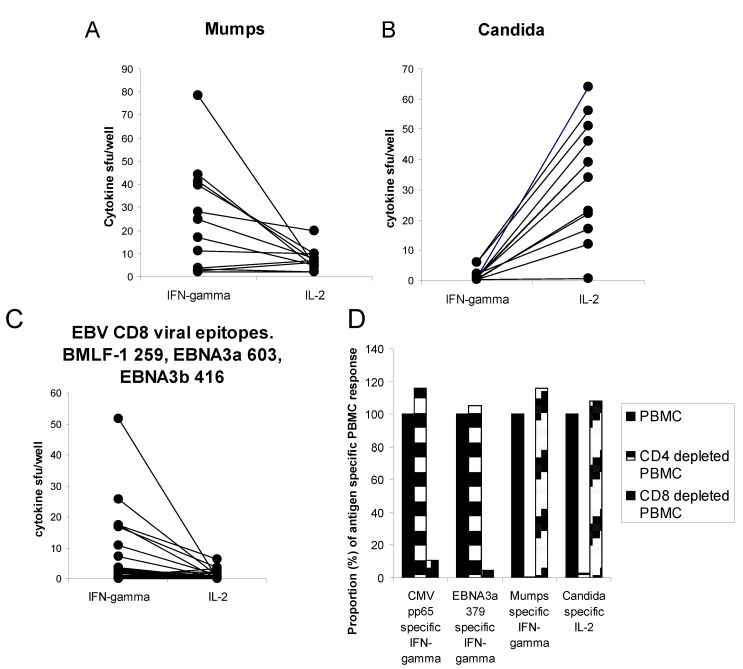
Antigen-specific CD4 and CD8 memory cells show dissociated production of IL-2 and IFN-γ. Healthy subject (n = 22) PBMC (**panels A–C**) or CD4 *vs.* CD8 depleted PBMC (**panel D**) (300,000 cells per well) were challenged in 20 h culture with recall protein (mumps or candida) or peptide (EBV BMLF-1, EBNA3a, or EBNA3b) antigen and IFN-γ/IL-2 producing cell frequency was measured by ELISPOT method. Assays were performed in triplicate. For **panels A–C** each line represents a separate subject (n = 12). **Panel D**, CD4 *vs.* CD8 depletion analysis reveals immune response phenotype. Spot forming units (sfu) observed with PBMC (300,000 cells per well) were assigned as 100%. Sfu observed when plating the same number of CD4 depleted PBMC or CD8 depleted PBMC are shown as a proportion (%) of PBMC activity.

To begin to explore the relation between IFN-γ and IL-2 producing T cells in a recall antigen specific response, we measured memory IFN-γ and IL-2 producing CD4 and CD8 T cell frequencies in the periphery of healthy individuals previously exposed to mumps (by immunization), candida (usual cutaneous exposure), or viral antigen (seropositive for these antigens). As shown in Figure 1, within each individual, and within the group as a whole, memory CD4 responses to mumps, and CD8 responses to viral antigen, were notable for greater IFN-γ than IL-2 secreting frequency, while the Candida specific IL-2 secreting memory CD4 T cell frequency was greater than that of the IFN-γ secreting frequency. Therefore, within a given individual, memory cells targeting one antigen may differ from those targeting another antigen.

**Figure 2:**
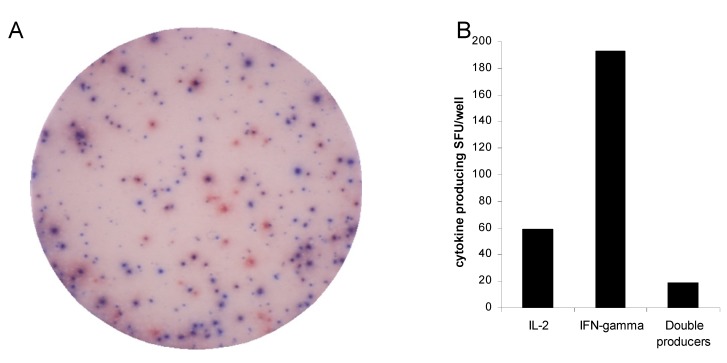
Functional heterogeneity of antigen specific immunity at the single cell level indicates IFN-γ producing memory cells usually do not produce IL-2. PBMC from a representative subject were challenged with mumps-antigen (1/80 dilution) in 20 h culture. 2-color ELISPOT assay was performed. Digital image of a representative well is shown in **panel A**. Blue spots represent IFN-γ producing cells while red spots represent IL-2 producing cells. Spot forming units are represented in bar graph format in **panel B**.

To evaluate the antigen specific T cell population in more detail, at the single T cell level, we evaluated IFN-γ and IL-2 secreting memory T cells using a 2-color ELISPOT assay. As shown in Figure 2, antigen specific cells that secrete IFN-γ usually do not secrete IL-2, and those that secrete IL-2 usually do not secrete IFN-γ in a 20 h ELISPOT assay. Therefore IFN-γ and IL-2 secreting function do not necessarily reflect the same T cell population

### 3.2. Allo-Antigen-Specific T Cells Show Dissociated IL-2-IFN-γ Production

**Figure 3:**
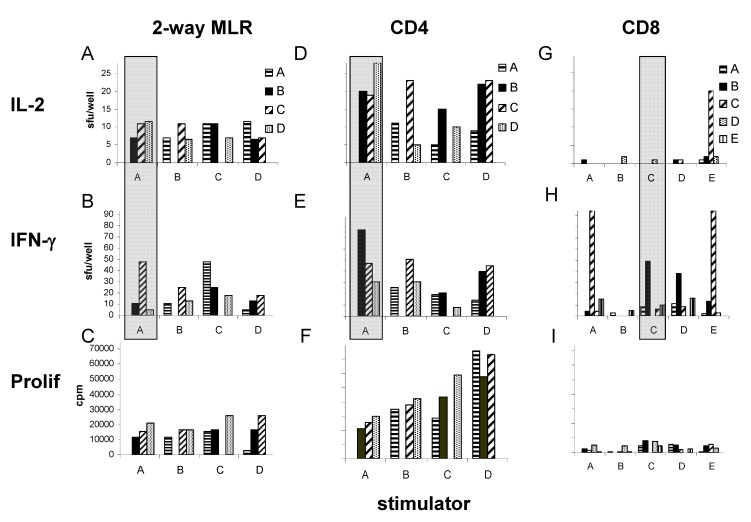
Allo-antigen-specific T cells show dissociated IL-2-IFN-γ production. Allogeneic 20 h cultures were performed as 2-way MLR with 3 × 10^5^ PBMC (**panels A–C**), or as a 1-way allogeneic response using CD3/CD56 depleted PBMC stimulators (300,000 cells/well) and CD4 (**panels D–F**), or CD8 (**panels G–I**) cell responders (300,000 cells/well). IL-2 sfu (**panels A, D, G**), IFN-γ sfu (**panels B, E, H**) and proliferation cpm (**panels C, F, I**) are shown. Stimulator identification is shown on the x-axis, while responder identification is shown in the legend. Shaded regions highlight discordance between IL-2 and IFN-γ producing effector function. Stimulator or responder cell alone control cultures resulted in <5 sfu and <1,000 cpm (not shown).

To explore the relation between IL-2 and IFN-γ production in the setting of the allogeneic T cell response, we specifically analyzed alloantigen specific IFN-γ and IL-2 producing frequencies in the repertoire of unfractionated lymphocytes, CD4 lymphocytes, and CD8 lymphocytes. As shown in Figure 3, when alloantigen specific T cell reactivity was explored among 5 healthy subjects (culturing PBMC or CD3-/56- PBMC stimulators with all possible combinations of PBMC, CD4 or CD8 T cell responders from the same donors) we observed significant IFN-γ and IL-2 producing alloreactive frequencies in 2-way **MLR** (unfractionated cell Mixed Lymphocyte Reactions, where both party T cells were capable of secreting cytokine; panels **A and B**). When the CD4 compartment was separately evaluated in 1-way alloreactive reactions, we observed no autoreactive T cells, but commonly observed both IFN-γ and IL-2 producing alloreactive cells in short term recall (Panels **D and E**). The time frame of this response is consistent with memory function, since naïve cells are incapable of secreting cytokine in this time frame [11,18,19]. When comparing IFN-γ and IL-2 producing frequency we observed both overlap and distinct differences. For example, highlighted alloreactive CD4 T cells from donor D reacted most vigorously with stimulator cells from donor A when IL-2 clonal size was evaluated (**Panel D,** highlighted), while alloreactive CD4 T cells from donor B reacted most vigorously with stimulator cells from donor A when IFN-γ clonal size was evaluated (**Panel E**, highlighted). Comparing magnitude of IFN-γ to IL-2 producing alloreactive T cell repertoire within the CD8 compartment, IL-2 producing alloreactive cells were infrequently detected, while IFN-γ secreting alloreactive cells were commonly observed (**panels G and H**). When we compared alloantigen specific IFN-γ and IL-2 producing frequency in the CD4 T cell fraction, IFN-γ producing frequency was found to be greater (mean 34.1 *vs.* 17.1 spot forming units (**sfu**), *p* = 0.003, Table 1). Therefore, in a polyclonal allo-specific recirculating memory T cell population, different subpopulations are capable of different effector function.

**Table 1:** IFN-γ frequency dominates during first 20 h of allogeneic response.

Stimulator × Responder	IL-2	IFN-γ
A × B	20	77
A × C	19	47
A × D	28	31
B × A	11	26
B × C	23	50
B × D	21	31
C × A	4	19
C × B	15	21
C × D	10	8
D × A	9	14
D × B	22	40
D × C	23	45
Mean *	17.1	34.1

Allogeneic CD4 T cell immunity was measured in 4 healthy subjects (A-D), using CD3/CD56 depleted PBMC stimulators (300,000/well), and CD4 T cell responders in 20 h culture. ELISPOT analysis was performed and IL-2 and IFN-γ sfu/well are shown. * IL-2 *vs.* IFN-γ mean sfu *p* = 0.003.

### 3.3. The Allogeneic Proliferative Response Correlates with the Frequency of IL-2, but not with IFN-γ Producing T Cells

We next evaluated the relation between proliferative activity, IFN-γ secreting activity, and IL-2 secreting activity in the allogeneic response. As shown in the bottom panels of Figure 3, proliferation, as measured by tritiated thymidine incorporation, was readily detectible over the course of 5 days. Overall, when alloreactive cytokine secreting CD4 T cells were observed within the first 20 h of the response, proliferative activity was commonly observed (**panels D–F**). However, the rank order magnitude of proliferative activity over 5 days did not always parallel the cytokine secreting frequencies over the first 20 h. When we specifically analyzed the relationship between proliferation, IFN-γ secretion, and IL-2 secretion we observed a significant correlation between IL-2 producing frequency during the first 20 h and proliferative activity over 5 days in the CD4 T cell fraction (r = 0.54, *p* < 0.05; Table 2). This relationship was strengthened when IL-2 producing CD4 frequency was evaluated at 3 days (r = 0.62, *p* < 0.01). In contrast, there was no significant relation between IFN-γ and proliferative activity in the CD4 T cell fraction. Moreover, in the CD8 T cell fraction, no relation between IFN-γ or IL-2 secreting frequency and proliferation was observed. In the case of CD8 T cell IL-2 secretion, the lack of a relationship was clearly due to the fact that we rarely observed IL-2 secreting CD8 T cells

**Table 2:** Correlations among IFN-γ, IL-2, and proliferation.

**Compared parameters**	2-way MLR	CD4 allogeneic response	CD8 allogeneic response
**20 h IL-2 *vs.* proliferation**	r = 0.59 *	R = 0.54 *	R = 0.24
**20 h IFN-γ *vs.* proliferation**	r = 0.52 *	R = 0.37	R = 0.23
**72 h IL-2 *vs.* proliferation**	r = 0.73 **	R = 0.62 **	ND
**72 h IFN-γ *vs.* proliferation**	r = 0.37	R = 0.43	ND

Correlation coefficients are shown for each comparison. ND = not done. * *p* < 0.05;** *p* < 0.01.

### 3.4. The Proliferative Response Entails a Substantial Non-T Cell Bystander Component

**Figure 4:**
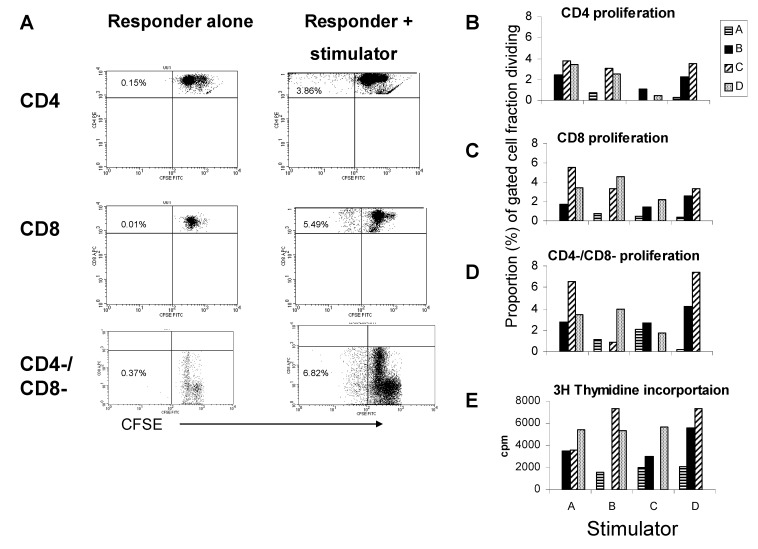
The allogeneic proliferative response entails a substantial non-T cell bystander component. Panel A. representative proliferative reaction by CFSE analysis. CFSE labeled allogeneic responder PBMC (300,000 cells/well) were cultured with CD3/56 depleted PBMC for 6 days. Lymphocyte gate was determined by forward and side scatter. CFSE dye dilution was analyzed on responder alone (left panels) *vs.* responder and stimulator (right panels) gating on CD4, CD8 and CD4-/CD8- cell fractions. Reactions for 4 separate stimulators (represented on *x*-axis) and responders (represented in legend) are shown in **panels B–E**, representing CD4 (**panel B**), CD8 (**panel C**), and CD4-/CD8- (**panel D**) proliferation by CFSE dye dilution analysis, or bulk proliferation by ^3^H thymidine incorporation (**panel E**).

**Figure 5:**
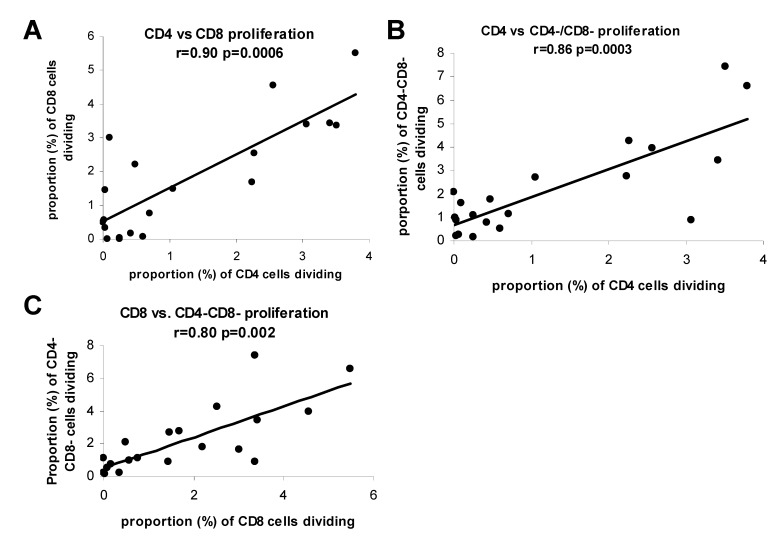
During mixed lymphocyte reactions soluble factors are likely involved with proliferation. Associations between CD4 T cell and CD8 T cell (**panel A**), between CD4 T cell and CD4-/CD8- cell (**panel B**), and between CD8 T cellCD4 T cell and CD4-/CD8- cell (**panel C**) proliferation as determined by CFSE dye dilution method for reactions described in Figure 4.

We next evaluated the relation between CD4 and CD8 T cell proliferation in the allogeneic response. Responder PBMC from 4 different donors were labeled with CFSE and reacted with allogeneic CD3/56 depleted PBMC from each of the same donors (in all possible combinations). After 6 days, alloreactive CD4, CD8, and CD4-/CD8- cell division was measured by flow cytometric analysis of CFSE dye dilution. As shown in Figure 4, in the presence of allogeneic stimulator cells (CD3/56 depleted PBMC) CD4, CD8, and CD4-/CD8- cell proliferation occurred (**panel A**). Notably, CD4, CD8, and CD4-/CD8- cell proliferative activities appeared to have the same rank order in magnitude for each allogeneic stimulator when all possible combinations of stimulator-responder pairings were evaluated (**panels B-D**). Additionally, tritiated thymidine incorporation rank order magnitude also appeared to match well with CFSE analysis of T cell subsets (compare **panel E** with **panels B-D**). When we specifically analyzed the relation among CD4, CD8, and CD4-/CD8- proliferation we observed striking correlations (Figure 5). In fact proliferative activities of all cell fractions (T and non-T cell) were directly related, consistent with a dependence of one activity on the other, or a common soluble factor. These findings suggest a bystander effect is operative in the allogeneic reaction.

**Figure 6:**
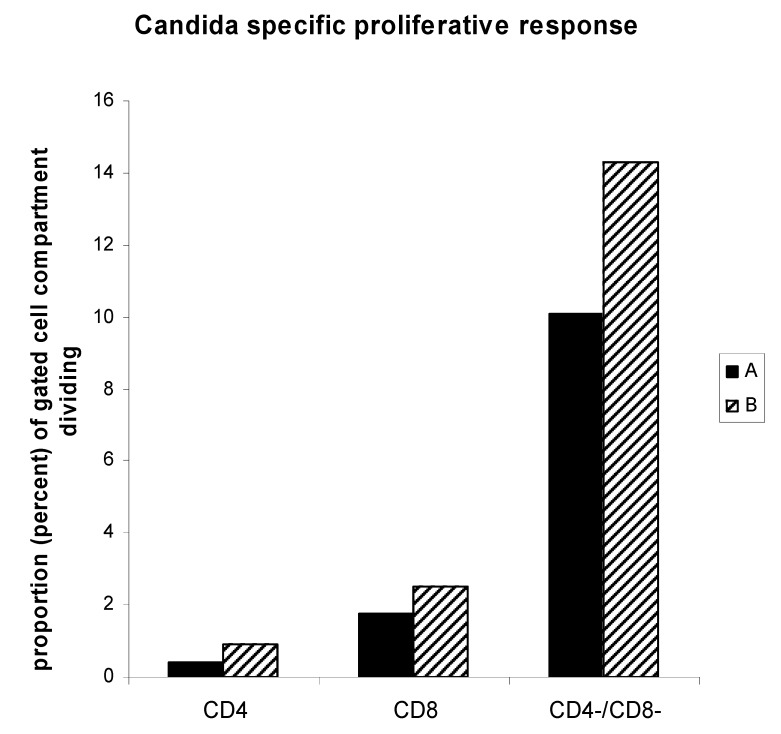
Soluble antigen specifc proliferative response also entails a substantial non-T cell bystander component. PBMC from two individuals with candida specific IL-2 and IFN-γ secreting CD4 T cell populations (identified in Figure 1 as CD4 T cell mediated activity) were analyzed by CFSE dye dilution analysis of 6 day antigen specific proliferation reactions. Analysis of CD4, CD8, and CD4-/CD8- cell fractions proliferating in response to soluble antigen are represented for each individual.

To determine whether non-T cell proliferation may exist in a single antigen specific T cell fraction, we selected 2 individuals with candida specific IL-2 and IFN-γ secreting CD4 T cell populations (Figure 6). Analysis of CD4, CD8, and CD4-/CD8- cell fractions proliferating in response to antigen revealed substantial proportions of CD8 and non-T cells dividing over the course of 6 days, even though these memory cell populations were contained within the CD4 T cell compartment when 20 h IFN-γ and IL-2 secreting function was analyzed (same subjects represented in Figure 1). Therefore, during an antigen specific proliferative response, bystander effect may also be operative.

## 4. Discussion

Upon activation, naïve T cells are capable of IL-2 production, while only memory T cells are capable of producing effector cytokines such as IFN-γ [18,20,21,22]. IL-2, as well as other soluble factors, secreted by naïve and memory T cells are thought to be critical for T cell expansion [18]. After initial antigen challenge, IL-2 producing memory T cells may exist as a precursor to effector cytokine producing memory T cells [13]. Further delineation of memory T cell subsets indicates that central memory T cells are capable of IL-2 and IFN-γ production, while effector memory T cells are capable of extremely rapid secretion of effector cytokines [9,10]. These fundamental, functionally distinct states of the memory T cell clonal mass are further reinforced by the known specialized nature of the antigen specific T cell at the single cell level; that is, each antigen specific T cell uncommonly produces more than one cytokine [20]. Here, when we analyzed antigen and allo-antigen specific T cell populations we uncommonly find IFN-γ and IL-2 co-expressing cells, and there is usually discordance between the secretion of these 2 cytokines at the single T cell level. When we did observe IL-2 production, proliferation was observed in an associated manner, indicating soluble factor likely plays a role in proliferation. Further, bystander non-T cell proliferation was also observed in the setting of antigen specific IL-2 production, with CD4, CD8 and non-T cell proliferation all associated. In the setting of clinical pre-transplant evaluation for potentially pathogenic T cells, evaluation of both CD4 and CD8 T cells capable of effector function offers potential for identifying a suboptimal donor-recipient pairing. Therefore, since IL-2 and IFN-γ producing function is often discordant, independent measurement of IFN-γ and other effector cytokine producing functions may be key in predicting clinical outcome after transplantation.

Antigen specific IL-2 producing T cells that do not secrete IFN-γ have been previously described [13,19]. Such cells are prevalently seen after immunizations with protein antigens, and are capable of further differentiation into effector cytokine secreting cells upon further antigen challenge. Here we also observe IL-2 producing memory CD4 T cells that do not secrete IFN-γ in direct *ex vivo* assays. In particular, Candida specific CD4 T cells more commonly secreted IL-2 as opposed to IFN-γ upon antigen re-encounter. This situation may in part be the result of mucosal surface antigen exposure, in the absence of adjuvant. In the allogeneic setting, IL-2 secreting CD4 T cells have previously been referred to as naïve CD4 T cells with allogeneic cross-reactivity. However, these cells may in fact be memory T cells. Truly naïve T cells in mice are not thought to make cytokine in the time frame of antigen exposure utilized in our assays [11,19,20]. The distinction between naïve and memory T cells with allogeneic reactivity may present different challenges in identification of immunomodulatory strategies. Further analysis of the susceptibility of these alloreactive populations to immunomodulatory agents in direct *ex vivo* assays will likely yield further insight into appropriate clinical strategies.

## Conclusions

Overall, it appears that proliferative responses may be reflective of soluble factor (including IL-2), bystander mediated effects during the allogeneic reaction. Because effector cytokines, such as IFN-γ, may play a different role in graft rejection, and because IL-2 and IFN-γ producing T cell populations are often discordant, it appears that proliferative readout alone may be incapable of predicting graft specific immune responses capable of mediating rejection. Indeed, pre-transplant IFN-γ reactivity to donor tissue has been found to correlate with subsequent graft rejection [1,4,5]. Our data here provide insight into the mechanism of why IFN-γ secretion may be selectively predictive, and why proliferative function using prior assay readouts such as thymidine incorporation may not be predictive. These data highlight the need for more careful analysis of donor specific immunity in the pre-transplant and post-transplant setting.
